# The INTERGROWTH-21^st^ Preterm Postnatal Growth Standards are robust across geographical settings and time

**DOI:** 10.1007/s00431-026-07494-1

**Published:** 2026-10-21

**Authors:** Francesca Giuliani, Robert B. Gunier, Alexandre Lapillonne, Maria Albertina S. Rego, Adele Winsey, Stephen A. Rauch, Paola Roggero, Maria Clara Restrepo Mendez, Sonia Deantoni, Montserrat Izquierdo Renau, Irma Alejandra Coronado Zarco, Shabina Ariff, Constanza Soto Conti, Roseline Ochieng, Funda Tuzun, Jacqueline Asibey, Chandrakala Bada Shekharappa, Gabriela Tavcioska, Leila Cheikh Ismail, Camilla Menis, Irina Ryumina, Ricardo Nieto, Josephine N. A. Agyeman-Duah, Brenda Frias Madrid, Marina Markelova, Brenda Eskenazi, Zulfiqar A. Bhutta, Aris T. Papageorghiou, Stephen H. Kennedy, Jose Villar

**Affiliations:** 1 Neonatal Special Care Unit, Regina Margherita Children Hospital, Turin, Italy; 2 Center for Environmental Research and Community Health, School of Public Health, University of California, Berkeley, USA; 3 Paris Cite University, APHP Hôpital Universitaire Necker-Enfants Malades, Paris, France; 4 Department of Pediatrics, Federal University of Minas Gerais, Belo Horizonte, Brazil; 5 Nuffield Department of Women’s & Reproductive Health, University of Oxford, Oxford, UK; 6 Neonatal Intensive Care Unit, Fondazione IRCCS Ca’ Granda Ospedale Maggiore Policlinico, Milan, Italy; 7 Neonatal Intensive Care Unit and Paediatric Department, Santa Croce Hospital, Moncalieri, Italy; 8 Neonatology Unit, Hospital Sant Joan de Deú, Barcelona, Spain; 9 Instituto Nacional de Perinatología “Isidro Espinosa de los Reyes”, Mexico City, Mexico; 10 Division of Woman & Child Health, Dept of Paediatrics and Child Health, The Aga Khan University, Karachi, Pakistan; 11 Division Neonatología, Hospital Materno Infantil Ramón Sarda, Buenos Aires, Argentina; 12 The Aga Khan Hospital, Nairobi, Kenya; 13 Department of Pediatrics, Division of Neonatology, Dokuz Eylul University Faculty of Medicine, Izmir, Turkey; 14 Holy Family Hospital, Brong Ahafo Region, Techiman, Ghana; 15 Neonatology Department, St. Johns Medical College Hospital, Bangalore, India; 16 General Hospital With Extended Activity Prilep, Prilep, North Macedonia UAE; 17 Department of Clinical Nutrition and Dietetics, College of Health Sciences, University of Sharjah, Sharjah, UAE; 18 Center for Obstetrics, Gynecology and Perinatology, Moscow, Russian Federation; 19 Center for Global Child Health, Hospital for Sick Children, Toronto, Canada; 20 Oxford Maternal & Perinatal Health Institute, Green Templeton College, University of Oxford, Oxford, UK

**Keywords:** Postnatal growth, Preterm infants, Growth standards

## Abstract

We investigated the robustness of the INTERGROWTH-21^st^ Preterm Postnatal Growth Standards for monitoring the postnatal growth of preterm infants a decade after the original study that produced them. The INTERPRACTICE-21^st^ Study assessed the early postnatal growth of all infants born preterm in 15 study sites across 14 countries. From this sample, we selected a ‘standard-like’ cohort of mother-newborn dyads as in the original study*.* Thus, we selected all singleton babies of healthy mothers, born between 27 and < 37 weeks’ gestation, without congenital malformations, fetal growth restriction or severe postnatal morbidity, i.e. confirming their eligibility for inclusion in the original standards*.* We used, across all study sites, the same standardised anthropometric measurements and feeding practices based on human milk. The main outcomes were the skeletal, fat-free mass indicators head circumference and length, following WHO recommendations. Variance component analysis and second-degree fractional polynomial regression models in a multi-level framework accounting for repeated measures were used to compare the growth trajectory of the ‘standard-like’ cohort to the standards. Of 3634 preterm babies enrolled, 1998 were singletons with complete birth anthropometric data. Among these, 1073 (54%) mother-baby dyads met the ‘standard-like’ eligibility criteria. The between-site differences in early postnatal growth of head circumference and length accounted for only 10.2% and 12.7% of the total variability of these repeated measures, proportions considerably lower than those for the total between individuals within a site variance of 80% and 79%, respectively. These low proportions supported pooling data across study sites and reinforced the consistency of the original results across geographical settings. *Conclusion*: The early growth of healthy preterm infants in the INTERPRACTICE-21^st^ ‘standard-like’ cohort, assessed a decade after the original study, closely matched the pattern originally described by the INTERGROWTH-21^st^ standards, which appear therefore robust to geographical changes and time.

**Table:** 

**What is Known:** • *The INTERGROWTH-21st Preterm Postnatal Growth Standards are the only international standards, based on the WHO prescriptive approach, for monitoring the growth of preterm infants. They were produced by following, from early pregnancy, uncomplicated preterm infants who were born to healthy mothers and achieved adequate growth and development at age 2.*	
**What is New:** *• In a study conducted in 14 countries worldwide, skeletal growth trajectories in a contemporary "standard-like" cohort of preterm infants demonstrated much larger within, than between, study site variance and closely matched the INTERGROWTH-21st standards, supporting the consistency of preterm growth across populations and over time.*

**Supplementary Information:**

The online version contains supplementary material available at https://doi.org/10.1007/s00431-026-07494-1.

The INTERGROWTH-21^st^ Consortium has described, in geographically diverse urban regions worldwide, using a range of biomarkers, how all humans should grow and develop from early pregnancy to 2 years of age. In the INTERGROWTH-21^st^Fetal Growth Longitudinal Study (FGLS), a large cohort of healthy, adequately-nourished, educated women with a naturally conceived singleton pregnancy was enrolled [1, 2]. We adopted the prescriptive methodology [3] of the World Health Organization (WHO) Multicentre Growth Reference Study (MGRS) [4], which produced the WHO Child Growth Standards [5]. Therefore, FGLS participants met rigorous, individual and population-level inclusion criteria, i.e. they were, at individual and population level, at low risk of adverse maternal and perinatal outcomes.

Infants born preterm (< 37 weeks’ gestation) in the FGLS cohort were enrolled in the Preterm Postnatal Follow-up Study (PPFS) and followed up prospectively to construct, for the first time, postnatal growth standards for preterm infants based on the actual follow-up of preterm newborns. We also demonstrated that they remained healthy with adequate growth and motor development up to 2 years of age, supporting their adequacy for constructing growth standards [6].

Their standardised postnatal measures of weight, length and head circumference contributed to international standards for the postnatal growth of preterm infants up to 64 post-menstrual weeks, which is when they match the WHO Child Growth Standards [7]. These postnatal standards have been adopted by multiple countries and regions worldwide and international agencies, including WHO and the Centers for Disease Control and Prevention (CDC) during the Zika epidemic.

However, from a biological and epidemiological perspective, it is important to consider whether these standards require revision in changing environments [8, 9], analogous to post-marketing surveillance.

To address this question, we analysed data from the INTERPRACTICE-21^st^Study, which investigated the postnatal growth of preterm infants, their feeding practices and early morbidity in 14 countries worldwide [10]. From this contemporary, highly standardised prospective cohort—enrolled approximately a decade after PPFS—we selected a sub-set of pregnancies and preterm newborns that would have met the eligibility criteria for inclusion in the original INTERGROWTH-21^st^ Preterm Postnatal Growth Standards [7]. We assessed the early postnatal growth of this ‘standard-like’ preterm cohort against those standards, using the same analytical strategy, study protocol and rigorously standardised measurement equipment, alongside feeding recommendations prioritising human milk. We present here the results of that comparison.

## Methods

The INTERPRACTICE-21^st^ Study was implemented between January 2018 and June 2020 in medical institutions in 15 cities in 14 countries: Buenos Aires, Argentina; Belo Horizonte, Brazil; Paris, France; Techiman, Ghana; Milan and Turin, Italy; Bangalore, India; Nairobi, Kenya; Prilep, North Macedonia; Mexico City, Mexico; Karachi, Pakistan; Moscow, Russia; Barcelona, Spain; Izmir, Turkey; and Oxford, UK. These institutions delivered standardised pregnancy and neonatal care and promoted evidence-based neonatal and infant feeding recommendations [11]. The main objectives of this multicentre study were to promote optimal postnatal growth of preterm infants until 6 months post-term and standardise growth measurement across multiple countries by implementing the INTERGROWTH-21^st^international standards and evidence-based feeding recommendations. The analyses presented are a secondary analysis of prospectively collected and standardized data from the INTERPRACTICE-21^st^ Study. As the analyses here were not part of the study’s hypotheses, all researchers, clinical staff and members of the Oxford Coordinating Unit were unaware of the comparisons being undertaken for the present work.

Standardised forms were used to collect data on maternal demographics, medical and pregnancy-specific conditions, delivery history, neonatal and infant anthropometry, feeding practices and comorbidities until hospital discharge. All neonatal diagnoses and treatments were standardised, as were the main neonatal care practices using a protocol based on evidence-based practices [12].

Gestational age was estimated by ultrasound measurement of crown-rump length at < 14^+0^ weeks’ gestation ^13^ or head circumference at < 24^+0^ weeks’ gestation using INTERGROWTH-21^st^ standards ^14^.

Anthropometric measurements were taken using PPFS protocols, training materials and quality control procedures [15]. Newborn length and head circumference were obtained within 12–24 h of birth using the same equipment at all sites. An infantometer (Harpenden, Chasmors Ltd, London, UK) was used to measure recumbent length at birth and weekly until hospital discharge. A metallic non-extendable tape (Chasmors Ltd), precise to 1 mm with an 8 cm blank lead-in, was used to measure head circumference at birth and weekly until hospital discharge. The trained study anthropometrists independently took all measures twice and compared values using maximum allowable differences of 7 mm for length and 4 mm for head circumference. If any difference exceeded those values, both observers independently performed the relevant measurement a second time and, if necessary, a third time. The Oxford Anthropometric Standardisation Unit regularly monitored staff performance and recommended retraining if measures consistently exceeded the maximum allowable differences.

### Sample selection to replicate the INTERGROWTH-21^st^ Preterm Postnatal Growth Standards

From the main INTERPRACTICE-21^st^ study, we selected the sub-set of preterm newborns that would have been eligible to contribute data to the INTERGROWTH-21^st^ Preterm Postnatal Growth Standards [7].

Thus, we excluded multiple pregnancy, women that smoked during pregnancy, were obese (body mass index (BMI) ≥ 30 kg/m[2]), had an ultrasound diagnosis of suspected fetal growth restriction or were alcohol/drug users. We included only newborns born ≥ 27 weeks gestation, without any severe neonatal morbidity as defined in our conceptual paper (i.e., bronchopulmonary dysplasia, hypoxic-ischaemic encephalopathy, sepsis, neonatal anaemia requiring transfusion, periventricular haemorrhage and leukomalacia) [16], and they were not small-for-gestational-age (z-score for birth weight or length above—2 standard deviations (SD) of the INTERGROWTH-21^st^Newborn Size Standards and Very Preterm Size at Birth Reference Charts [17, 18]). We excluded newborns with preterm-associated impaired fetal growth, who were likely to be diagnosed with extrauterine growth restriction, as well as newborns with measures > 5 SD of the mean of the overall gestational-age specific values considered outliers, as was done in the original study. We did not exclude women that had fertility treatment or those who were underweight (BMI < 18.5 kg/m^2^), but we performed a sensitivity analysis to check that neither parameter influenced the results.

For the comparison with the median postnatal growth curves, we truncated the measures of infants born ≤ 33 weeks’ gestation at 38 weeks’ postmenstrual age because most healthy infants had been discharged by that stage, so the remaining measures were largely from preterm infants with associated severe morbidities.

In summary, for the present analyses, we included preterm newborns that could have been eligible to contribute to the preterm postnatal growth standards’ population, i.e. neonates without congenital malformations, fetal growth restriction or severe postnatal morbidity, born after uncomplicated pregnancies.

### Statistical analysis

The statistical approach was based on the WHO recommended methodology used in the original study: first, justify pooling the postnatal data from all 15 sites given the very low between-sites variability in skeletal size using the WHO definition [2, 19], and second, the same statistical strategy used to construct the INTERGROWTH-21^st^ Preterm Postnatal Growth Standards [7].

We applied variance component analysis to calculate the percentage of variance between-sites and within-sites from repeated measures of the same two fat-free mass, skeletal measures (head circumference and length). These measures are recommended as the best option to compare growth among populations [4] because they are resistant to skewing in response to excessive nutrition, as opposed to weight or other fat-related indicators; they are normally distributed (unlike fat-related indicators); are more precise than fat-related measures and are used to compare populations in the WHO MGRS thereby providing conceptual continuity with our study. We used the criteria of intraclass correlation coefficient above 0.75 (excellent correlation) for pooling measurements across sites [20].

We applied to the complete sample a multi-level, linear regression analysis, adjusting for postmenstrual age, which was treated as a fixed effect, whereas sites and individuals were treated as random effects [21].

The best-fitting powers for the median postnatal growth were provided by second-degree fractional polynomials and further modelled in a multi-level framework to account for the study’s longitudinal design [22, 23]. To obtain an equation for the SD, we modelled the resulting variance components from the multi-level model that accounts for the between- and within-subject correlations using fractional polynomials. In all cases, we applied the fractional polynomial smoothing technique [22]. The data structure is composed of two levels, i.e. measures-within subjects, so we fitted a random-effects model (2-level hierarchical structure) to the longitudinal postnatal measures as a function of postmenstrual age using the *runmlwin* package in STATA [24].

Sex- and gestational age-specific *z*-scores were estimated for head circumference and length using the international INTERGROWTH-21^st^ Preterm Postnatal Growth Standards [7]. Gestational age at birth was tested in exploratory fractional polynomial models, was not statistically significant and was not therefore included in the final model.

We conducted sensitivity analyses excluding women that were either taking fertility treatments or who were underweight as these represent different eligibility criteria from the original cohort. All postnatal results are presented in exact weeks according to ‘postmenstrual’ age. All analyses were performed in STATA, version 17.0, software (StataCorp LP, College Station, TX, USA). All documentation was introduced into the specially developed data management system for entering data online locally (www.medscinet.com).

The INTERPRACTICE-21^st^ Study was approved by the Oxfordshire Research Ethics Committee ‘C’ (reference: 08/H0606/139), the research ethics committees of the individual participating institutions and the corresponding regional health authorities in which the project was implemented. Participants provided written informed consent to be involved in the study. The research was carried out in accordance with the Declaration of Helsinki.

## Results

**Fig. 1:**
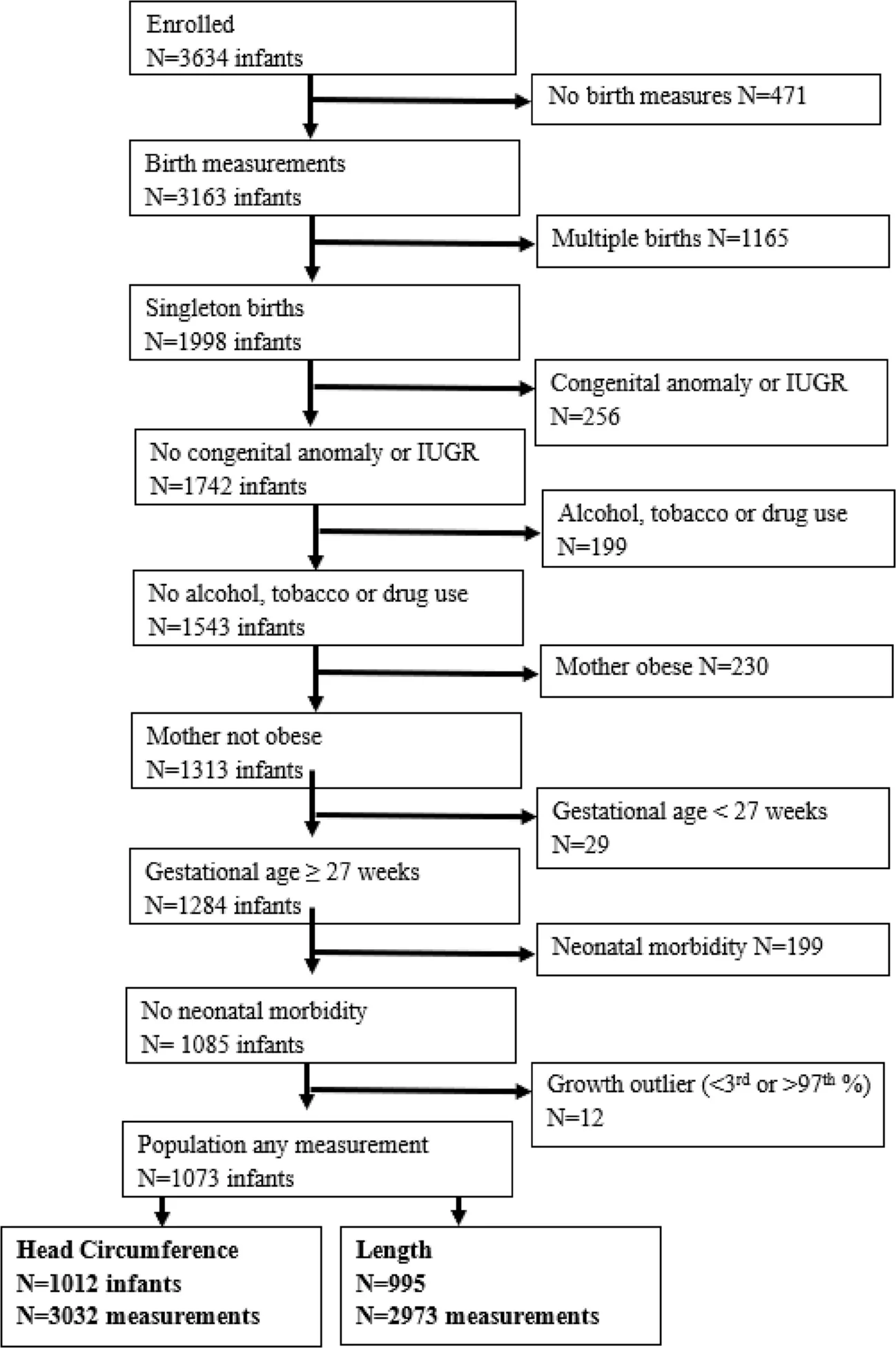
INTERPRACTICE-21^st^Study participant flowchart

A total of 3634 preterm babies, born alive between 23^+0^ and 36^+6^ weeks’ gestation to mothers > 18 years of age, were enrolled across the 15 sites and monitored from birth to hospital discharge. We excluded 1165 twin births and 471 newborns because of missing birth measures. The approximately identical eligibility criteria for enrolment in PPFS were then applied to the remaining 1998 singleton newborns and their associated pregnancies, which resulted in a cohort of 1313 newborns, of whom 1284 (97.8%) were born ≥ 27 weeks’ gestation. Finally, we excluded 199/1284 (15.5%) newborns as they developed severe early neonatal morbidity (defined as per protocol and consistent with our conceptual publication [16]), and 12/1284 (0.9%) defined as outliers in at least one neonatal anthropometric measure. This left a sample of 1012 infants with a total of 3032 head circumference measures and 995 infants with a total of 2973 length measures (Fig. 1) for inclusion in the present study, that would have been eligible for the original cohort which was used to construct the INTERGROWTH-21^st^ standards, i.e. the ‘standard-like’ INTERPRACTICE-21^st^ cohort.

Table 1 shows the maternal and newborn characteristics of this ‘standard-like’ INTERPRACTICE-21^st^ cohort. The mean (± SD) maternal age and BMI were 30.9 (6.1) and 23.6 (3.3) kg/m^2^, respectively. Most women (91.8%) were married or co-habiting and 47.0% were nulliparous. Compared to the original PPFS cohort (*n* = 201), the women in the ‘standard-like’ INTERPRACTICE-21^st^ cohort (*n* = 1073) were slightly older, more multiparous and had a higher caesarean section rate (59.0% v. 39.8%) in line with global trends. In addition, a higher percentage of the new cohort’s newborns were admitted to a Neonatal Intensive Care Unit (NICU) for more than 1 day (57.4% v. 40.8% in the original cohort).

**Table 1:** Maternal characteristics and neonatal events: INTERGROWTH-21^st^ Preterm Postnatal Follow-up Study (PPFS) and INTERPRACTICE-21^st^ (IP21) ‘standard-like’ cohort

	PPFS	IP21
	*N* = 201 *N* (%) or M ± SD	*N* = 1073 *N* (%) or M ± SD
Maternal age (years) (SD)	28.5 (4.1)	30.9 (6.1)
Maternal height (cm) (SD)	162.0 (5.8)	161.4 (7.2)
Maternal pre-pregnancy weight (kg) (SD)	61.9 (9.2)	61.5 (9.6)
Body mass index (kg/m^2^) (SD)	23.5 (3.1)	23.6 (3.3)
Gestational age at first antenatal visit (weeks) (SD)	11.7 (1.4)	15.4 (7.6)
Years of formal education (SD)	14.6 (2.9)	13.8 (4.3)
Haemoglobin level < 15 weeks’ gestation (g/dl) (SD)	12.4 (1.1)	12.3 (1.3)
Married/cohabiting (%)	192 (95.5)	979 (91.8)
Nulliparous (%)	146 (72.6)	503 (47.0)
Gestational age at birth (weeks) (SD)	35.5 (1.7)	34.5 (2.2)
Caesarean section (%)	80 (39.8)	631 (59.0)
NICU admission > 1 day (%)	82 (40.8)	614 (57.4)
Low birth weight (< 2500 g) (%)	108 (53.7)	660 (61.6)
Male sex (%)	99 (49.3)	562 (52.4)
Exclusive breastfeeding at discharge (%)	145 (72.1)	705 (65.8)
Birth weight (g) (SD)	2452 ± 519	2297 ± 552
Birth length (cm) (SD)	45.6 ± 2.7	44.7 ± 3.5
Birth head circumference (cm) (SD)	31.7 ± 1.8	31.4 ± 2.2

*NICU* Neonatal Intensive Care Unit, *M* mean, *SD* standard deviation

The mean (± SD) gestational age at birth in the INTERPRACTICE-21^st^ and PPFS cohorts was 34.5 (2.2) and 35.5 (1.7) weeks, respectively. The distribution of preterm gestational ages at birth in the two cohorts was 315 (29.4%) and 28 (13.9%) at 33 weeks or less, 425 (39.6%) and 68 (33.8%) at 34–35 weeks, and 333 (31.0%) and 105 (52.2%) at 36 weeks to less than 37 weeks; 213 (19.9%) and 12 (6.0%) neonates were born very preterm (27–32 weeks), respectively. However, the differences in the proportional distribution of gestational age at birth are a consequence of the over-sampling of very preterm infants in the INTERPRACTICE-21^st^ protocol. The prevalence of exclusive breastfeeding at hospital discharge in the two cohorts was 65.8% and 72.1%, respectively.

**Fig. 2:**
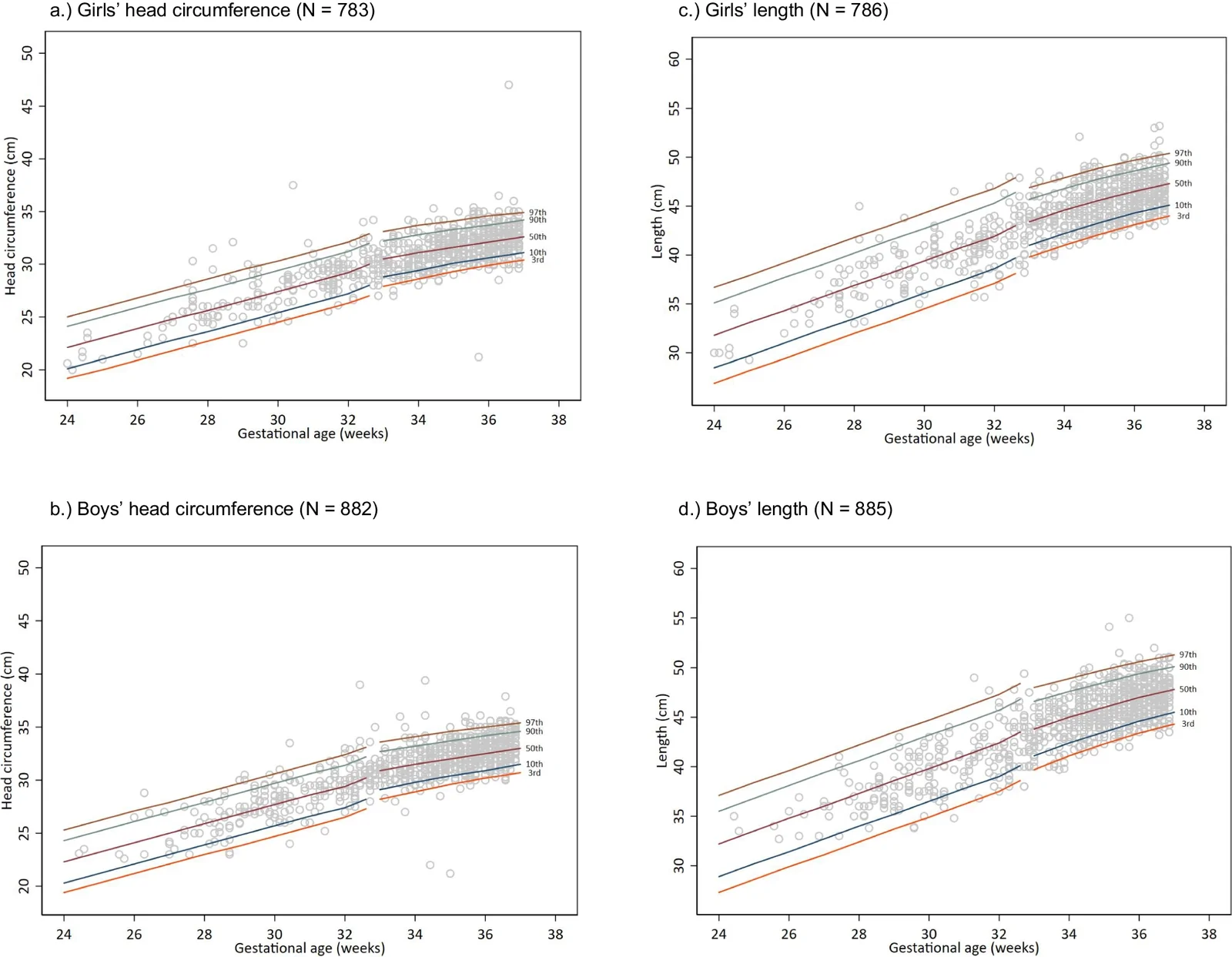
Distribution of birth head circumference and length in cm by gestational age in infants born preterm (< 37 weeks’ gestation) in the FGLS-like sample of the INTERPRACTICE-21^st^ Study. Note: Only measures within 5 SDs of the mean of the overall gestational-age specific values were included.  Lines show the 3rd, 10th, 50th, 90th and 97th centile curves of INTERGROWTH-21st international standards for newborn head circumference according to gestational age. Note: Only measures within 5 SDs of the mean of the overall gestational-age specific values were included. Lines show the 3rd, 10th, 50th, 90th and 97th centile curves of INTERGROWTH-21st international standards for newborn length according to gestational age

The mean birth length was lower in the INTERPRACTICE-21^st^cohort, in line with the lower mean gestational age (44.7 v. 45.6 cm), but the mean head circumference was similar. We further explored if the differential baseline characteristics between the two cohorts could have influenced size at birth as a key pregnancy outcome related to postnatal growth. Overall, the birth head circumference and length according to gestational age and sex of the INTERPRACTICE-21^st^ cohort were compatible with the corresponding INTERGROWTH-21^st^ Newborn Size at Birth Standards, and the Very Preterm Size at Birth Reference Charts (Fig. 2) [17, 18]. In sensitivity analyses, excluding women either taking fertility treatment or who were underweight, the mean birth measures were nearly identical to the overall population (Supplemental Table 1).

For head circumference and length, the two measures of skeletal growth selected a priori following the same strategy as in the original study, we conducted variance component analysis to estimate the proportions of total variance between sites and between individuals within a site, as per protocol [2, 20]. The values for between sites and individuals (Table 2) were 10.2% and 80.0% for head circumference, and 12.7% and 78.9% for length, respectively. Hence, the small contribution of between-site differences to the total variance (intraclass correlation 0.86–0.89), compared to the large between-individuals within a site proportion, supported pooling all the sites’ data creating a single cohort to compare postnatal growth patterns with the original PPFS cohort [20].

**Table 2:** Variance component analysis for repeated measures of head circumference and length from preterm infants enrolled in the INTERPRACTICE-21^st^ ‘standard-like’ cohort

Variance component	Head circumference		Length	
	(*N* = 1012 infants; *n* = 3032 measurements)		(*N* = 995 infants; *n* = 2973 measurements)	
	Estimate (standard error)	Proportion (%)	Estimate (standard error)	Proportion (%)
Variance between sites	0.46 (0.22)	10.2	1.37 (0.64)	12.7
Variance between individuals within a site	3.60 (0.17)	80.0	8.49 (0.40)	78.9
Residual variance	0.44 (0.01)	9.8	0.91 (0.03)	8.4

Estimations are adjusted for age and sex as fixed effects; ‘study site’ and ‘individuals’ treated as random effects

**Fig. 3:**
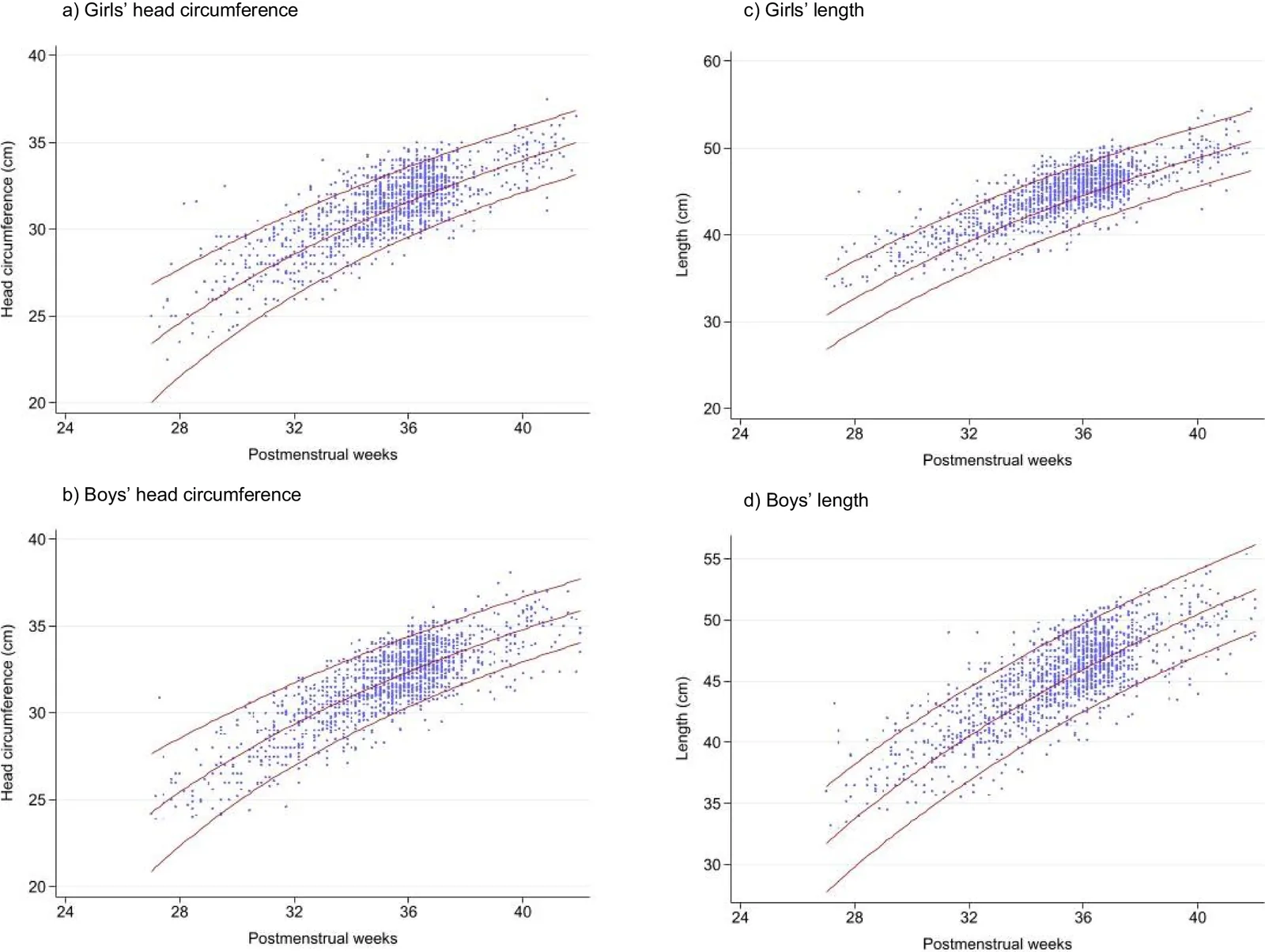
Scatterplot of postnatal head circumference and length measures for the INTERPRACTICE-21^st^ ‘standard-like’ cohort against the INTERGROWTH-21^st^ Preterm Postnatal Growth Standards. Boys and girls shown separately

**Fig. 4:**
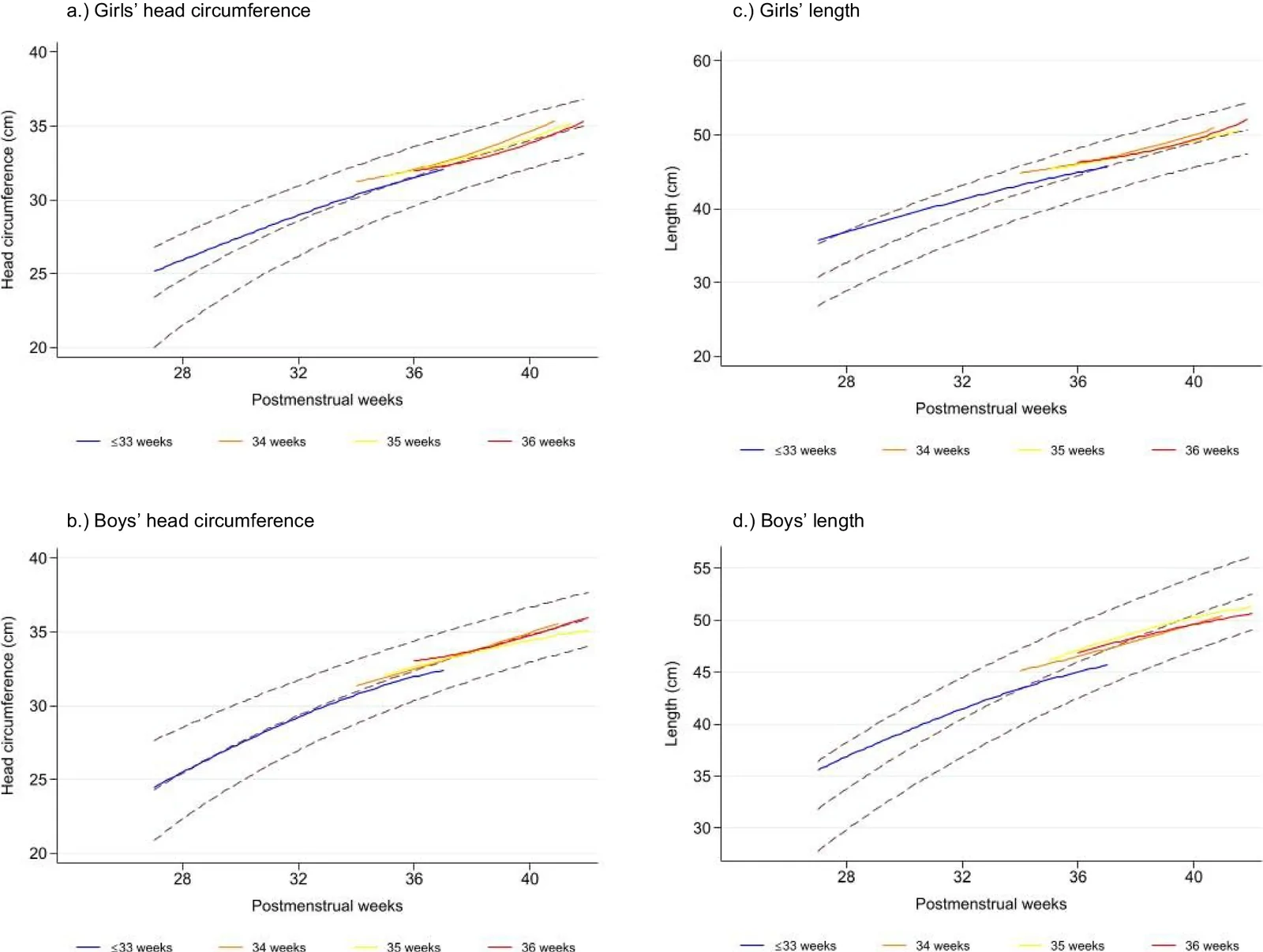
Postnatal head circumference and length stratified by sex and gestational age in the INTERPRACTICE-21^st^ Preterm Postnatal Follow-up Study

The raw data from 29 to 42 postmenstrual weeks are shown as scatter plots in Fig. 3 against the INTERGROWTH-21^st^ Preterm Postnatal Growth Standards for boys and girls separately. Furthermore, Fig. 4 shows the superimposed fitted 50th centiles for the two skeletal measures according to gestational age at birth. The fitted curves for the four strata of gestational age at birth follow the 50th centile lines from the original PPFS cohort and are very close together.

At 37 postmenstrual weeks, the mean difference between the 50th centile of all the gestational age at birth cohorts combined and each of the different gestational age cohorts was 0·16 cm for head circumference and 0·31 cm for length. These values are equivalent to 0·14 units of the SD of all cohorts combined at that age for head circumference and 0·15 SD for length. Thus, the separately fitted curves differ minimally from the curve fitted to the complete preterm cohort.

**Fig. 5:**
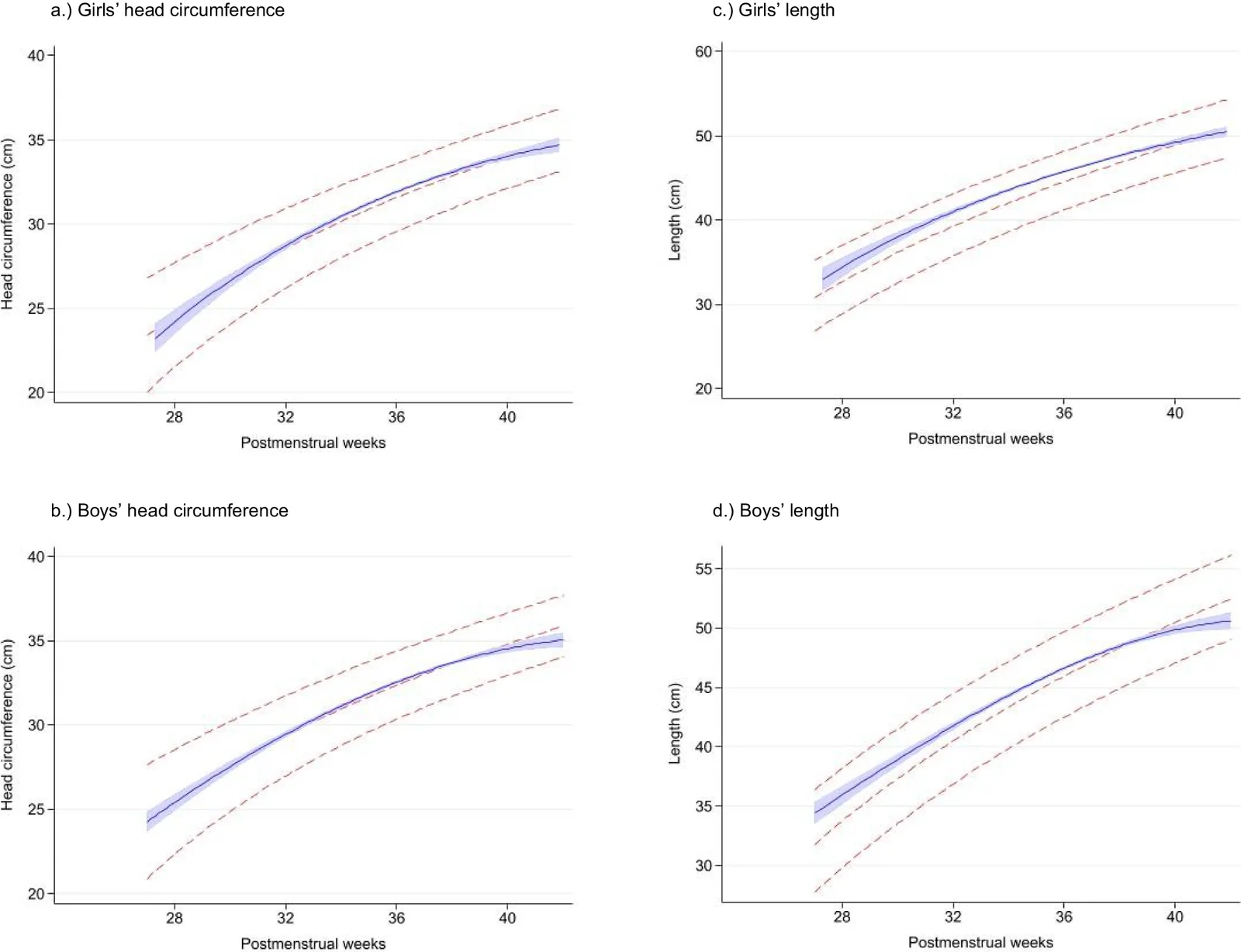
50^th^ centiles for postnatal head circumference and length, adjusted for gestational age at birth, for the INTERPRACTICE-21^st^ ‘standard-like’ cohort against the INTERGROWTH-21^st^ Preterm Postnatal Growth Standards. Boys and girls shown separately

Finally, Fig. 5 shows the 50th centile of postnatal growth trajectories up to hospital discharge for head circumference and length, adjusted for gestational age at birth, for boys and girls separately, plotted against the 3rd, 50th and 97th centiles of the original PPFS cohort that created the INTERGROWTH-21^st^ Preterm Postnatal Growth Standards [7]. The growth patterns for the head circumference measures are identical to the corresponding 50th centile of the standards and very similar for postnatal length trajectories (Fig. 5).

## Discussion

We have compared the early postnatal growth of two prospective international cohorts of preterm infants enrolled approximately 10 years apart to address the reliability of the INTERGROWTH-21^st^ Preterm Postnatal Growth Standards across time and in different geographical settings. Infant enrolment in both cohorts followed the same a priori conceptual framework [16], and the study protocols were almost identical. Each incorporated accurate ultrasound dating of pregnancy, excluded complicated pregnancies with inadequately grown fetuses and followed standardised neonatal care and feeding practices based on the use of human milk [11].

There are two key findings with relevant clinical and biological implications: the first is that a decade after PPFS, across study sites that were mostly different, only 10% of the total variability in longitudinal head circumference and length was attributable to between-site differences, far smaller than the 80% of the total variability from between-individuals within a site. The second is that the skeletal growth of ‘healthy’ or uncomplicated (for their expected maturation level) preterm infants, selected across a wide range of geographical settings using the same prescriptive approach recommended for growth monitoring, and whose neonatal care and feeding (which included the use of human milk) were standardised, followed approximately the same trajectory described in the INTERGROWTH-21^st^ Preterm Postnatal Growth Standards [7].

The slightly higher postnatal length (but not head circumference) values seen in Figs. 4 and 5 from 28 to 29 weeks’ gestation, may reflect, in addition to the greater variability and measurement error inherent in assessing length in very small preterm infants, advances in respiratory care and nutritional support for infants born at these very low gestational ages. These improvements have occurred alongside a shift from a predominantly incubator-based, technology-driven model toward a more evidence-based, family-centred approach.

It is important to reiterate that the INTERGROWTH-21^st^ Preterm Postnatal Growth Standards are robust, with adequate sample size, for monitoring the postnatal growth of 90% of preterm infants, i.e. moderate and late preterm. The postnatal growth of very preterm infants is not an issue related to the type of standard to be used, rather a therapeutic and nutritional dilemma that needs to be tested by comparing different feeding and care regimens in a large, multicentre, international randomised control trial.

The comparison conducted is robust for the aim of the present analysis and represents a unique contribution to the literature on growth monitoring following the introduction of a new clinical tool because (1) we followed an a priori conceptual framework and WHO recommendations for monitoring growth [25]; (2) all methods, equipment, training and quality control processes were standardised across sites; (3) data were collected specifically for both studies, i.e. we did not use routinely collected clinical data; (4) the infants’ intrauterine growth showed no evidence of fetal growth restriction, the leading factor for extrauterine growth faltering; (5) feeding practices were standardised and exclusive breastfeeding promoted and (6) the analytical approach, which was consistent with WHO recommendations [4], was identical to that used in all INTERGROWTH-21^st^ studies [19].

Importantly, we focused for comparison on skeletal indicators, which in the case of early postnatal growth are head circumference and length. We selected them because they are as follows: (1) resistant to skewing in response to overnutrition, contrary to fat-related indicators; (2) normally distributed (unlike fat-related indicators) and (3) supported by WHO recommendations and were used to compare populations for the WHO Child Growth Standards, currently used worldwide. The observation that only close to 10% of the total variability in longitudinal head circumference and length was attributable to between-site differences, is in agreement with those reported for fetal growth [2], postnatal length in term infants [5] and child height [26] and justified pooling the data to create international standards.

There are limitations associated with this type of ‘post marketing’ comparisons: (1) the maternal and neonatal characteristics of the two cohorts were not entirely similar, despite the approximately identical eligibility criteria. For example, in the INTERPRACTICE-21^st^ cohort, maternal age, caesarean section rate and the proportion of newborns admitted to NICU for more than 1 day, were all higher, whilst nulliparity and mean birth weight were lower. These modest differences in baseline characteristics are not unexpected when enrolling a much larger sample size from a wider range of free-standing geographical settings that can have a variety of underlying risk profiles, similar to a drug post-marketing exercise; (2) FGLS mothers were prospectively enrolled at < 14 weeks’ gestation and their infants, if born preterm and uncomplicated, were then enrolled in PPFS. In the ‘standard-like’ INTERPRACTICE-21^st^ Study, we applied approximately the same maternal and newborn eligibility criteria but to a sample of preterm infants and their mothers prospectively enrolled at birth and (3) we specifically oversampled lower gestational ages at birth to satisfy critics of the small sample size of very preterm newborns, but this, inevitably, has created at very low gestational ages, a ‘standard-like’ cohort at proportionally higher risk for comparative purposes. Nevertheless, the anthropometric measures at birth, were compatible with the INTERGROWTH-21^st^ Newborn Size at Birth Standards, and the Very Preterm Size at Birth Reference Charts [17, 18]. Hence, the fetal growth patterns according to gestational age at birth were adequate.

In summary, the cohort selected using approximately the same eligibility criteria as the original PPFS population had adequate size at birth, largely followed recommended feeding practices, and had a very small contribution of between-site differences to the total variance for postnatal skeletal growth justifying pooling the data. Consequently, the ‘standard-like’ INTERPRACTICE-21^st^ preterm cohort selected would have been eligible to contribute to the original cohort used to construct the INTERGROWTH-21^st^ Preterm Postnatal Growth Standards [7]. The new cohort’s growth trajectories were consistent with those standards, demonstrating their robustness across geographical location and over time, adding support to the use of international preterm postnatal growth standards.

## Supplementary Information

Below is the link to the electronic supplementary material.

ESM 1 DOCX (19.0 KB)

## Data Availability

Anonymized data will be made available upon reasonable request for academic use and within the limitations of the informed consent. Requests must be made to the corresponding author. Every request will be reviewed by the INTERPRACTICE-21^st^Consortium Executive Committee. After approval, the researcher will need to sign a data access agreement with the INTERPRACTICE-21^st^ Consortium.

## Declarations

### Disclaimer

The views expressed herein are those of the authors and not necessarily those of the NHS, the NIHR the Department of Health or any of the other funders. All other authors declare no conflicts.
