# Supplementary material for: The INTERGROWTH-21st Preterm Postnatal Growth Standards are robust across geographical settings and time
Source: Eur J Pediatr. 2026 Oct 21;185(11):870. doi: 10.1007/s00431-026-07494-1 (PMC13687238; doi:10.1007/s00431-026-07494-1)
Supplement: Supplementary file 1 [file 431_2026_7494_MOESM1_ESM.docx]

**Supplemental Table 1.** Sensitivity analyses of birth measures excluding those women taking fertility treatments and those with a low body mass index.

| Population | N | Birth Head Circumference (cm) | N | Birth Length (cm) |  |
| --- | --- | --- | --- | --- | --- |
|  |  | Mean ± SD |  | Mean ± SD |  |
| Overall | 1069 | 31.4 ± 2.2 | 1070 | 44.7 ± 3.5 |  |
|  |  |  |  |  |  |
| Exclude fertility treatment | 995 | 31.4 ± 2.2 | 996 | 44.7 ± 3.4 |  |
| Exclude BMI | 1002 | 31.4 ± 2.2 | 1003 | 44.8 ± 3.4 |  |
| < 18.5 kg/m^2^ |  |  |  |  |  |

Abbreviations: BMI = body mass index, SD = standard deviation.
